# Bacteridia and Malignant Pustule

**Published:** 1865-04

**Authors:** William Budd

**Affiliations:** The Manor House, Clifton


### MISCELLANEOUS.

### BACTERIDIA AND MALIGNANT PUSTULE.

To the Editor of The Lancet:

Sir:—The facts related in the following extract from a masterly article on Spontaneous Generation, by M. Jamin, in the *Revue des Deux Mondes*, are in all ways so interesting, that I make no apology for asking you to publish them. I ought to add that the italics which occur in one or two places are mine:

"Dr. Davaine has devoted himself for some years to the careful study of a terrible malady of the charbon genus—the splenic apoplexy (*sang de rate*—anglicée, 'blood') which develops itself spontaneously in sheep, and is inevitably fatal to them. The blood of the diseased animals, examined under the microscope, has been found crowded with minute organisms allied to the *bacteria*, and which have been named *bacteridia*. This blood injected into the tissue of another animal carries these creatures with it, and death is certain. The malady is equally transmitted when a rabbit is made to swallow either the blood or part of an animal affected with splenic apoplexy. The infected blood may be dried and *kept for an ndefinite time without losing the germs of the infusoria which it contains;*

and whenever it comes to be injected or to be given as food, the disease is propagated. These facts being ascertained, as the symptoms of splenic apoplexy offer some affinity to those of another malignant malady known by the name of 'charbon' (or malignant pustule,') inquiries were instituted as to whether there might not be a still closer bond between the two affections. 'Charbon' begins by a 'malignant pustule' of blackish color, surrounded by a ring of vesicles, which must be speedily destroyed by caustic, if a general infection is to be avoided. On the 14th of April of the present year (1864) Dr. Raimbert was called to a carter who had contracted a true malignant pustule on a farm where the sheep were suffering from splenic apoplexy. He removed the pustule, dried it at once, and handed it over to Dr. Davaine, who examined it under the microscope. It was a perfect *felt, composed entirely of bacteridia.* Rabbits fed with it contracted splenic apoplexy in consequence, and died with their blood crowded with *bacteridia,* and communicated 'charbon' to other animals. Here, then, is a disease transmitted from sheep to man, and appearing in him under the form of a pustule, which in its turn has the power of communicating to all animals the particular virus which it contains. And what is this virus? A brood of infusoria of a special and venomous species. *The smallest quantity suffices to kill, because it suffices to sow and multiply the species.* The malady is transmitted by inoculation, because the animalcules pass from the infected to the inoculated subject; it is transmitted by the air, because the germs dry up and are wafted away, and become again sown; possibly also, as many hold, by the bites of flies, which thus become the vehicle for the transmission of the *bacteridia.* Such is the explanation, not less simple than certain, of the effects of a particular virus. The future will decide how far it is possible to extend to all analogous cases so fertile a theory, but already it is easy to understand the hopes of physiologists and to predict their success; perhaps we are on the eve knowing, avoiding, and curing contagious scourges."

The facts here detailed are not altogether new. Virchow, and some earlier observers whose names escape me for the moment, had already pointed out the occurrence, in countless numbers, of a kind of "vibro" in the blood of *living* animals affected with charbon.

I have not been able to refer to Dr. Davaine's own account of these researches; but before the case which he wishes to make out for the minute organisms he describes can be considered as finally established, other data will be required beyond those adduced by his reviewer. Not only must the constant presence of this particular species of *bacteridia* in the diseases in question be ascertained, but its absence in other putrefactive disorders. In all such cases there is a special danger, which those who have most studied the subject will best appreciate, of falling into the old error of taking for essential what may possibly be only an epi-phenomenon. The perfect way in which the facts seem to explain all the conditions, although a strong argument in favor of the interpretation set upon them, may on the other hand, easily beguile us into a too ready acquiescence in it.

At the same time the whole tendency of recent research, and of Pasteur's discoveries in particular, is to the effect that the tribe of minute organisms to which the *bacteridia* belong, in reality take the initiative in, and are the primary cause of, the zymotic changes with which they are found associated.

The uncontrollable *itching* which marks the first stage of malignant pustule, and is so characteristic of it, is, when considered as a phenomenon which betrays the presence of so many parasites in other parts not undeserving of attention in connexion with Dr. Davaine's view.

Should his discovery be confirmed by more extended researches, it is one of which it will be difficult to overrate the value.

As regards malignant pustule its importance will be supreme. Diagnosis, pathology, origin, mode of propagation, and indications of cure, will be all summed up in the conditions which attach to the growth and multiplication of a single parasitic organism.

In relation to diagnosis, the fact is one which might eventually become of the greatest possible use. For if it be true that the first breed of *bacteridia* is developed in the part which is to be the seat of the future pustule, the practitioner, armed with the microscope and with the little "harpoon" with which the Germans dip for trichina, might ascertain the characteristic presence of these minuter parasites by means of an operation not more formidable than the puncture of a grooved needle.

But, as M. Jamin rightly suggests, the interest of this discovery, should it be confirmed, culminates in its relation to the subject of contagion generally.

In a memorandum on the Investigation of Epidemic and Epizootic Disorders, which I drew up at the request of the British Medical Association in March, 1863, there occurs the following passage:

"In order to render the inquiry on which the Association is about to enter really comprehensive, it would be necessary to associate with the study of epidemics that of the diseases caused in man and animals by living parasites, external and internal.

A fuller knowledge of the phenomena attaching to the dissemination of the prolific and minute germs of these parasites could not fail to be of great use in helping to the true interpretation of the phenomena which attach to the strictly analogous dissemination of the equally prolific and equally minute germs of contagious poisons.

In particular, it would be of the highest value in showing, by data that could not be gainsaid, what is the real worth of the negative evidence now so implicitly relied on, as an indication of spontaneous origin, and as opposed to the law of propagation by continuous succession.

Additional reasons for putting the parasites and the contagions together in such an inquiry are—1, that at many points the two blend insensibly one into the other; 2, that, with the advance of knowledge, diseases are constantly being transferred from the group of common contagions to the group of parasites; and, 3, that there already exists amongst the most advanced thinkers on these topics, a shrewd suspicion that the two groups will eventually coalesce, and be found to be in their essence identical."

Dr. Davaine's interesting discovery seems not unlikely to offer a striking illustration of more than one of the several positions here taken.

I am, Sir, your obedient servant,

William Budd, M. D.

The Manor House, Clifton, Feb. 5th, 1865.

